# Effectiveness of Email Warning on Reducing Hospital Employees’ Unauthorized Access to Protected Health Information: A Nonrandomized Controlled Trial

**DOI:** 10.1001/jamanetworkopen.2022.7247

**Published:** 2022-04-13

**Authors:** John Xuefeng Jiang, Nick Culbertson, Ge Bai

**Affiliations:** 1 Broad College of Business, Michigan State University, East Lansing; 2 Protenus, Inc, Baltimore, Maryland; 3 Johns Hopkins Carey Business School, Baltimore, Maryland; 4 Johns Hopkins Bloomberg School of Public Health, Baltimore, Maryland

## Abstract

This nonrandomized controlled trial evaluates whether email warnings for employees who access protected health information are associated with a reduction in subsequent unauthorized access.

## Introduction

Data breaches of protected health information (PHI) create substantial financial, reputational, and clinical risks for patients and health care entities.^1,2,3,4^ Prior research^5^ found that large academic medical centers face disproportionately higher PHI breach risks than other hospitals. Approximately one quarter of PHI breaches were caused by employees’ unauthorized access to PHI, in which the employee lacked authorization, permission, or other legal authority to access the data.^6^ A nonrandomized controlled trial was conducted in a large academic medical center to understand the effectiveness of email warning on reducing repeated unauthorized access to PHI.

## Methods

This study was exempt from institutional review board approval from Michigan State University because it does not meet the criteria for human participants research (no identifiable private information or identifiable biospecimens were accessed). The study followed the Transparent Reporting of Evaluations With Nonrandomized Designs (TREND) reporting guideline.

**Figure 1.**
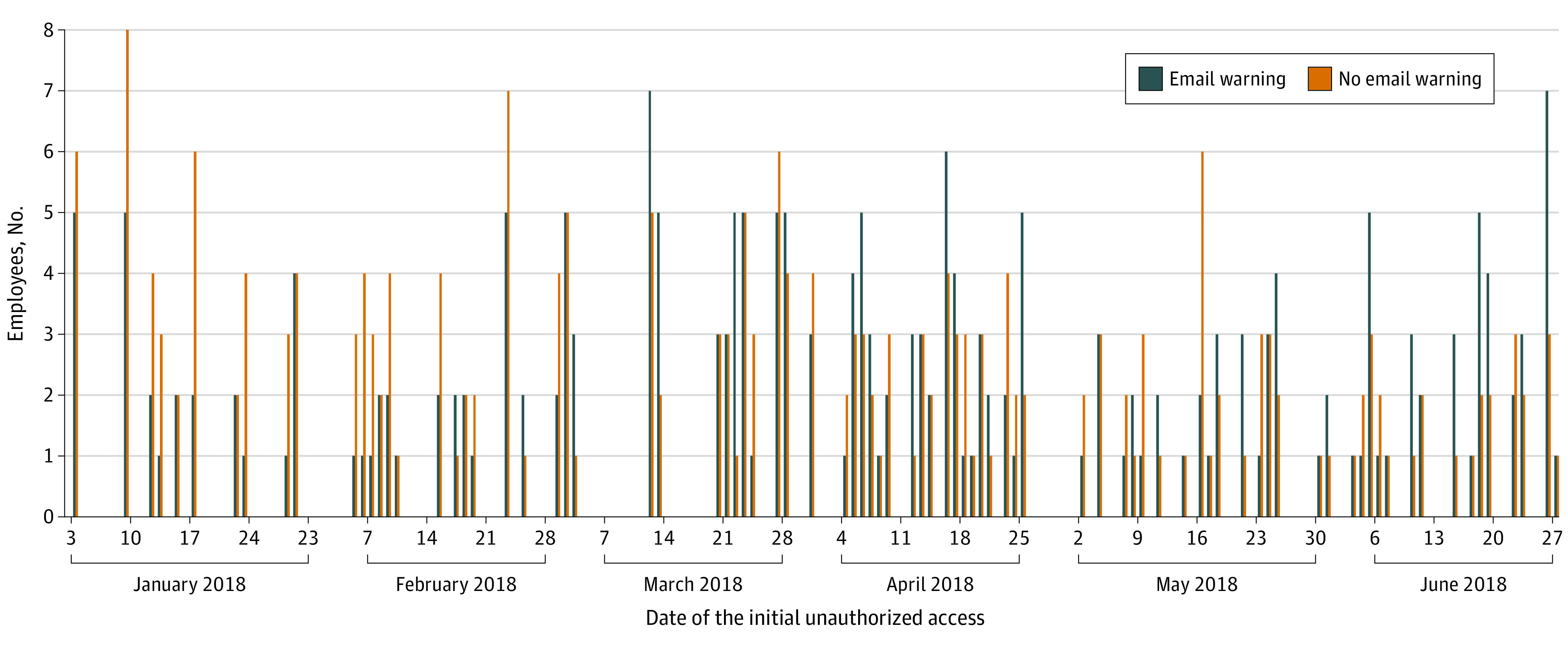
Frequency of Employees’ Initial Unauthorized Access, by Email Warning Status A total of 444 employees who committed unauthorized access were flagged. Approximately half of the employees (219) received an email warning on the day of the incident (intervention group), while the remaining 225 employees who committed unauthorized access on the same day did not receive an email warning (control group). Days without a bar had no unauthorized access occurrence.

From January 1 to July 31, 2018, a large academic medical center’s PHI access monitoring system flagged all unauthorized accesses to patient electronic medical records from 444 employees (all professional medical staff), who were not part of the patient’s intervention team and did not have access permission. A total of 219 employees (49%) were randomly selected to receive an email warning on the night of their access, while the remaining employees (225, 51%) served as controls (Figure 1). The email stated that the employee had been identified as having accessed a patient’s electronic medical record without a known work-related purpose and that unauthorized access is a privacy violation.

For the intervention group (receiving an email warning) and the control group (not receiving an email warning), the frequency of subsequent unauthorized access for the same employee during January 1 to July 31, 2018, was compared. All unauthorized access was later verified as valid PHI breaches (neither work-related nor patient-authorized).

The academic medical center prohibits employees from accessing the records of family members, coworkers, friends, or other acquaintances without prior written authorization. To preserve the trial’s validity, no disciplinary action was taken during the trial period. Upon the conclusion of the trial, disciplinary actions were taken on all identified offenders following the institution’s access policy.

The 2-sided *t* test was used to compare medians in unreimbursed Medicaid costs of nonprofit and for-profit hospitals. Statistical significance was set at *P* < .001. Statistical analysis was conducted using SAS statistical software version 9.4 (SAS Institute). Data were analyzed from August to December 2021.

## Results

**Figure 2.**
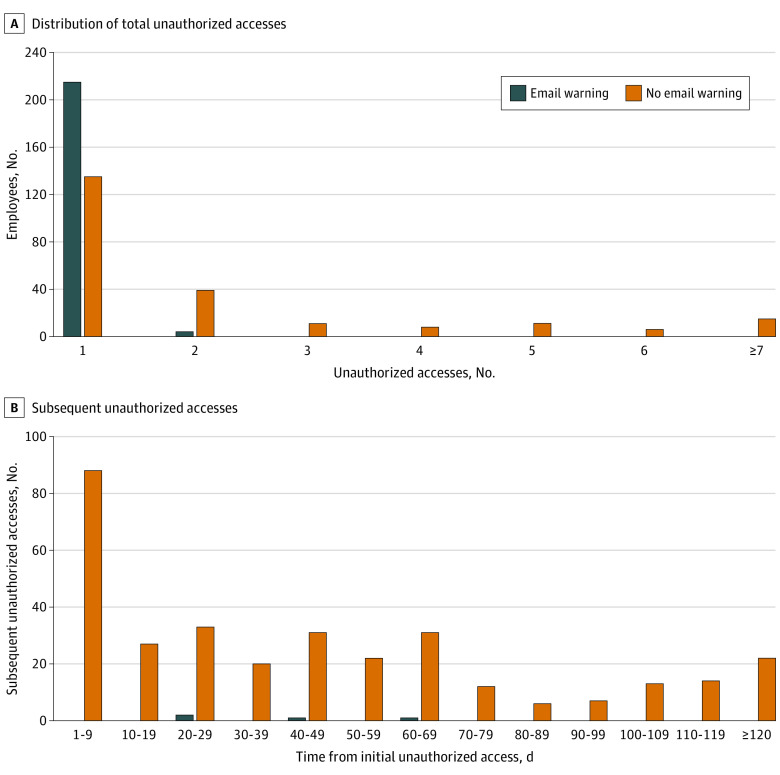
The Effectiveness of Email Warning A, A total of 444 employees who committed unauthorized access were flagged. Approximately half of the employees (219) received an email warning on the day of the incident (intervention group), while the remaining 225 employees who committed unauthorized access on the same day did not receive an email warning (control group). B, There were 4 subsequent unauthorized access incidents from the intervention group (all involving the same patient as the initial violation) and 326 incidents from the control group (313 involving the same patient as the initial violation).

A total of 444 employees accessed data for which they were not part of the patient’s intervention team and did not have access permission. From January 1 to July 31, 2018, only 4 of the 219 employees (2%) in the intervention group committed unauthorized access for a second time (Figure 2A), while 90 of the 225 employees (40%) in the control group did so, representing a 95% effectiveness of email warning in reducing repeated offenses (2% vs 40%). The mean frequency of unauthorized access was 1.02 in the intervention group vs 2.45 in the control group (difference, 1.43; 95% CI, 1.01-1.85; P < .001). In the intervention group, 4 repeated offenses occurred between 20 and 70 days after the initial unauthorized access (Figure 2B). In the control group, 326 repeated violations occurred, with 88 (27%) within 10 days after the initial unauthorized access and 56 (17%) after 90 days.

## Discussion

This nonrandomized controlled trial found that when left unchecked, hospital employees repeatedly committed unauthorized access to PHI, creating substantial financial, reputational, and clinical risks for the patient and the organization.^1^ Avoiding repeated access is a critical measure for risk mitigation. Email warning after initial unauthorized access is 95% effective in preventing repeated unauthorized access to PHI. Email warning remains a critical access control measure for the medical center today.

The results of this study might not be completely generalizable to other settings. The study is also limited by the lack of data on the prevalence of using email warning to contain unauthorized access among hospitals. Adopting simple email warnings, accompanied by a PHI access control system, can substantially reduce future unauthorized access and benefit patients and health care entities. The constantly evolving landscape of PHI breaches requires continuous risk management effort.
